# Association of CDK15 and L1CAM Expression in Cervical Cancer Tissues with Clinicopathological Characteristics and Recurrence/Metastasis After Radical Hysterectomy

**DOI:** 10.12669/pjms.42.3.13277

**Published:** 2026-03

**Authors:** Jingjing Li, Yuanyuan Su, Limin Chen, Yan An, Lin Shen

**Affiliations:** 1 Jingjing Li Department of Obstetrics and Gynecology, Affiliated Hospital of Hebei University, Baoding 071000, Hebei, China; 2 Yuanyuan Su Department of Obstetrics and Gynecology, Affiliated Hospital of Hebei University, Baoding 071000, Hebei, China; 3 Limin Chen Department of Obstetrics and Gynecology, Affiliated Hospital of Hebei University, Baoding 071000, Hebei, China; 4 Yan An Department of Obstetrics and Gynecology, Affiliated Hospital of Hebei University, Baoding 071000, Hebei, China; 5 Lin Shen Department of Obstetrics and Gynecology, Baoding Maternal and Child Health Hospital, Baoding 071000, Hebei, China

**Keywords:** Cervical Cancer, CDK15, Clinicopathological Characteristics, L1CAM, Recurrence and Metastasis

## Abstract

**Objective:**

To investigate the association of CDK15(cyclin-dependent kinase 15) and L1CAM L1 (cell adhesion molecule) expression with clinicopathological characteristics and recurrence and/or metastasis after radical hysterectomy of cervical cancer.

**Methodology:**

Retrospective analysis was performed on the clinical data of 80 patients with pathologically confirmed cervical cancer who underwent radical hysterectomy at Affiliated Hospital of Hebei University between January 2023 to January 2025. Differences in CDK15 and L1CAM L1 expression in cervical cancer tissues were compared across subgroups stratified by different clinicopathological characteristics, including age and International Federation of Gynecology and Obstetrics(FIGO) stage. Recurrence and metastasis in cervical cancer patients were documented within 12 months post-radical hysterectomy. The Kaplan-Meier method was employed to analyze the association of CDK15/L1CAM expression with recurrence-free survival(RFS).

**Results:**

The positive expression rates of CDK15 and L1CAM in cervical cancer tissues were 51.25% and 47.50%, respectively-both significantly higher than the corresponding rates of 22.50% and 18.75% in adjacent non-cancerous tissues(P<0.05). In cervical cancer tissues, the expression of CDK15 exhibited statistically significant differences across subgroups stratified by FIGO stage, histological differentiation, lymph node metastasis, and lymphovascular invasion(P<0.05); whereas the expression of L1CAM demonstrated statistically significant differences across subgroups stratified by FIGO stage, maximum tumor diameter, and lymph node metastasis(P<0.05). Kaplan-Meier analysis revealed that the 12 months RFS rate after radical hysterectomy was approximately 68.29% in cervical cancer patients with positive CDK15 expression, which was lower than the 89.74% in patients with negative CDK15 expression(Log-rank P=0.019).

**Conclusion:**

CDK15 and L1CAM are highly expressed in cervical cancer tissues. Consequently, CDK15 and L1CAM hold promise as novel prognostic biomarkers and potential therapeutic targets for cervical cancer.

## INTRODUCTION

With the widespread implementation of cervical cancer screening, expanded administration of human papillomavirus (HPV) vaccines, and refinement of surgical techniques, the incidence and mortality of cervical cancer in China have exhibited a declining trend. Nevertheless, approximately 15%–20% of patients still develop recurrence and metastasis following radical hysterectomy.^1^ The 5-year survival rate of patients with recurrence and metastasis following radical hysterectomy for cervical cancer is less than 30%.

Additionally, existing clinicopathological parameters (e.g., International Federation of Gynecology and Obstetrics [FIGO] staging, lymph node status) fail to accurately characterize tumor biological behavior, posing constraints on individualized prognostic prediction.^2^ With the advancement of molecular biology, a growing body of studies has centered on the role of tumor-associated molecular biomarkers in the progression and prognosis of cervical cancer. Cyclin-dependent kinase 15 (CDK15), a novel cyclin-dependent kinase, accelerates G1/S phase transition via phosphorylation of retinoblastoma (Rb) protein and thus suppresses cellular apoptosis; its aberrant expression has been confirmed to be associated with invasion and metastasis of various solid tumors, including lung cancer and breast cancer.^3^,^4^ L1 cell adhesion molecule (L1CAM) is a member of the immunoglobulin superfamily and was initially identified to be involved in axon guidance within the nervous system.

In recent years, emerging evidence has demonstrated that L1CAM promotes tumor cell migration and invasion by activating transcription factors associated with the ERK/MAPK and PI3K/AKT signaling pathways, and its association with lymph node metastasis in endometrial cancer has been validated.^5^ Currently, most studies on cervical cancer biomarkers focus on traditional proliferation markers, such as HPV-related proteins and Ki67, while research on novel molecular biomarkers (e.g., CDK15 and L1CAM L1) remains limited. This study aimed to explore the expression profiles of CDK15 and L1CAM L1 in cervical cancer tissues and the association of these expressions with recurrence and metastasis following radical hysterectomy, with the aim of providing a novel molecular basis for risk stratification and individualized treatment of cervical cancer patients after radical hysterectomy.

## METHODOLOGY

Retrospective analysis was conducted on the clinical data of 80 patients with pathologically confirmed cervical cancer who underwent radical hysterectomy at Affiliated Hospital of Hebei University between January 2023 to January 2025. The evaluation process of patients’ outcomes was double-blind. Patients were stratified into the recurrence/metastasis group and non-recurrence/metastasis group based on the presence of recurrence and metastasis within 12 months after radical hysterectomy for cervical cancer. Criteria for assessing recurrence and metastasis: New lesions were detected via imaging modalities(e.g., pelvic CT, gynecological ultrasound[B-mode]) or pathological examinations, and the pathological type of the new lesions was histologically consistent with that of the primary tumor.

### Ethical approval:

The study was approved by the Institutional Ethics Committee of Affiliated Hospital of Hebei University (No.:HDFYLL-KY-2023-052; date: March 14, 2023), and written informed consent was obtained from all participants.

### Inclusion criteria:

- Aged 28 to 70 years
- Patients with pathologically confirmed cervical cancer.
- Those who underwent radical hysterectomy plus pelvic lymph node dissection.
- Those with FIGO stage^6^ IA-IIB.
- Those with complete pathological records and follow-up data.

### Exclusion criteria:

- Patients with a comorbidity of other malignant tumors or a past history of malignant tumors.
- Those with preoperative distant metastasis or postoperative follow-up duration of < 12 months.
- Those with severe autoimmune diseases, hepatic or renal failure, or other underlying diseases that might impact prognosis.
- Those who died of non-cervical cancer-related causes during follow-up.

### Detection of CDK15 and L1CAM expression:

Reagents included the following: mouse anti-human CDK15 monoclonal antibody, mouse anti-human L1CAM monoclonal antibody(Santa Cruz Biotechnology, Inc., Dallas, USA), and streptavidin-peroxidase(S-P) hypersensitive immunohistochemistry(IHC) kit (Fuzhou Maixin Biotech Co., Ltd., Fuzhou, China). Surgically resected cervical cancer tissues and adjacent non-cancerous tissues (≥5 cm from the tumor margin) were sectioned into slices with a thickness of approximately 4 μm, and CDK15 and L1CAM L1 expression was detected via IHC. For result interpretation, five non-overlapping representative fields were selected under 400× magnification. The proportion of positive cells and staining intensity were quantified separately, and the mean values were used for semi-quantitative analysis.

Scoring criteria for positive cell proportion: 0 points were assigned for no positive cells, one point for positive cells <1/3, two points for positive cells 1/3–2/3, and 3 points for positive cells >2/3. Scoring criteria for staining intensity: 0 points were assigned for no staining, one point for light yellow, 2 points for tan, and 3 points for dark brown. A product of the positive cell proportion score and staining intensity score ≥3 was defined as positive expression (this criterion applied to both CDK15 and L1CAM L1). For quality control, two pathologists with intermediate or higher academic titles reviewed the slides in a double-blind fashion. If the score discrepancy exceeded one, the two pathologists jointly reviewed the slides to reach a consensus. All the patients followed up for one year.

### Clinicopathological characteristics:

The clinicopathological characteristics of patients included age, FIGO staging, pathological type, maximum tumor diameter, histological differentiation grade, lymph node metastasis, and lymphovascular invasion.

### Statistical analysis:

Data were analyzed using SPSS 26.0 software, with the statistical significance level set at P<0.05. Count data(expressed as *n*, %) were compared using the chi-square(*χ²*) test. Recurrence-free survival(RFS) was estimated using the Kaplan-Meier method. Independent prognostic factors were identified using the Cox proportional hazards regression model.

## RESULTS

The positive rates of CDK15 and L1CAM L1 expression in cervical cancer tissues were 51.25% and 47.50%, respectively, which were significantly higher than the corresponding 22.50% and 18.75% in adjacent non-cancerous tissues (all P<0.05) Table-I.

**Table-I:** CDK15 and L1CAM L1 Expression Between Cervical Cancer Tissues and Adjacent Non-Cancerous Tissues (n, %).

Type of Cervical Tissue	Number of Cases	Positive CDK15	Positive L1CAM
Cancer tissue	80	41 (51.25)	38 (47.50)
Adjacent non-cancerous tissue	80	18 (22.50)	15 (18.75)
*χ* ^2^		14.204	14.925
*P*		<0.05	<0.05

In cervical cancer tissues, CDK15 expression exhibited statistically significant differences across subgroups stratified by FIGO stage, histological differentiation, lymph node metastasis, and lymphovascular invasion (all P<0.05); whereas L1CAM L1 expression demonstrated statistically significant differences across subgroups stratified by FIGO stage, maximum tumor diameter, and lymph node metastasis (all P<0.05) Table-II and III.

**Table-II:** Differences in CDK15 Expression in Cervical Cancer Tissues Stratified by Clinicopathological Characteristics (n, %).

Clinicopathological Characteristic	Number of Cases	CDK15		χ^2^	P
		Positive (n=41)	Negative (n=39)		
Age				0.075	0.784
≥50 years	32	17 (41.46)	15 (38.46)		
<50 years	48	24 (58.54)	24 (61.54)		
FIGO stage				5.263	<0.05
IA~IB	56	24 (58.54)	32 (82.05)		
IIA~IIB	24	17 (41.46)	7 (17.95)		
Pathological type				0.765	0.382
Adenocarcinoma	25	11 (26.83)	14 (35.90)		
Squamous cell carcinoma	55	30 (73.17)	25 (64.10)		
Maximum tumor diameter				0.055	0.814
≥4 cm	38	20 (48.78)	18 (46.15)		
<4 cm	42	21 (51.22)	21 (53.85)		
Histological differentiation degree				4.984	<0.05
Poor differentiation	26	18 (43.90)	8 (20.51)		
Moderate to high differentiation	54	23 (56.10)	31 (79.49)		
Lymph node metastasis				12.041	<0.05
Yes	32	24 (58.54)	8 (20.51)		
No	48	17 (41.46)	31 (79.49)		
Lymphovascular invasion				8.542	<0.05
Yes	38	26 (63.41)	12 (30.77)		
No	42	15 (36.59)	27 (69.23)		

**Table-III:** Differences in L1CAM Expression in Cervical Cancer Tissues Stratified by Clinicopathological Characteristics (n, %).

Clinicopathological Characteristic	Number of Cases	L1CAM		χ^2^	P
		Positive (n=38)	Negative (n=42)		
Age				0.134	0.715
≥50 years	32	16 (42.11)	16 (38.10)		
<50 years	48	22 (57.89)	26 (61.90)		
FIGO stage				10.397	<0.05
IA~IB	56	20 (52.63)	36 (85.71)		
IIA~IIB	24	18 (47.37)	6 (14.29)		
Pathological type				0.179	0.673
Adenocarcinoma	25	11 (28.95)	14 (33.33)		
Squamous cell carcinoma	55	27 (71.05)	28 (66.67)		
Maximum tumor diameter				9.709	<0.05
≥4 cm	38	25 (65.79)	13 (30.95)		
<4 cm	42	13 (34.21)	29 (69.05)		
Histological differentiation degree				0.622	0.430
Poor differentiation	26	14 (36.84)	12 (28.57)		
Moderate to high differentiation	54	24 (63.16)	30 (71.43)		
Lymph node metastasis				12.707	<0.05
Yes	32	23 (60.53)	9 (31.43)		
No	48	15 (39.47)	33 (78.57)		
Lymphovascular invasion				0.764	0.382
Yes	38	20 (52.63)	18 (42.86)		
No	42	18 (47.37)	24 (57.14)		

**Fig.1:**
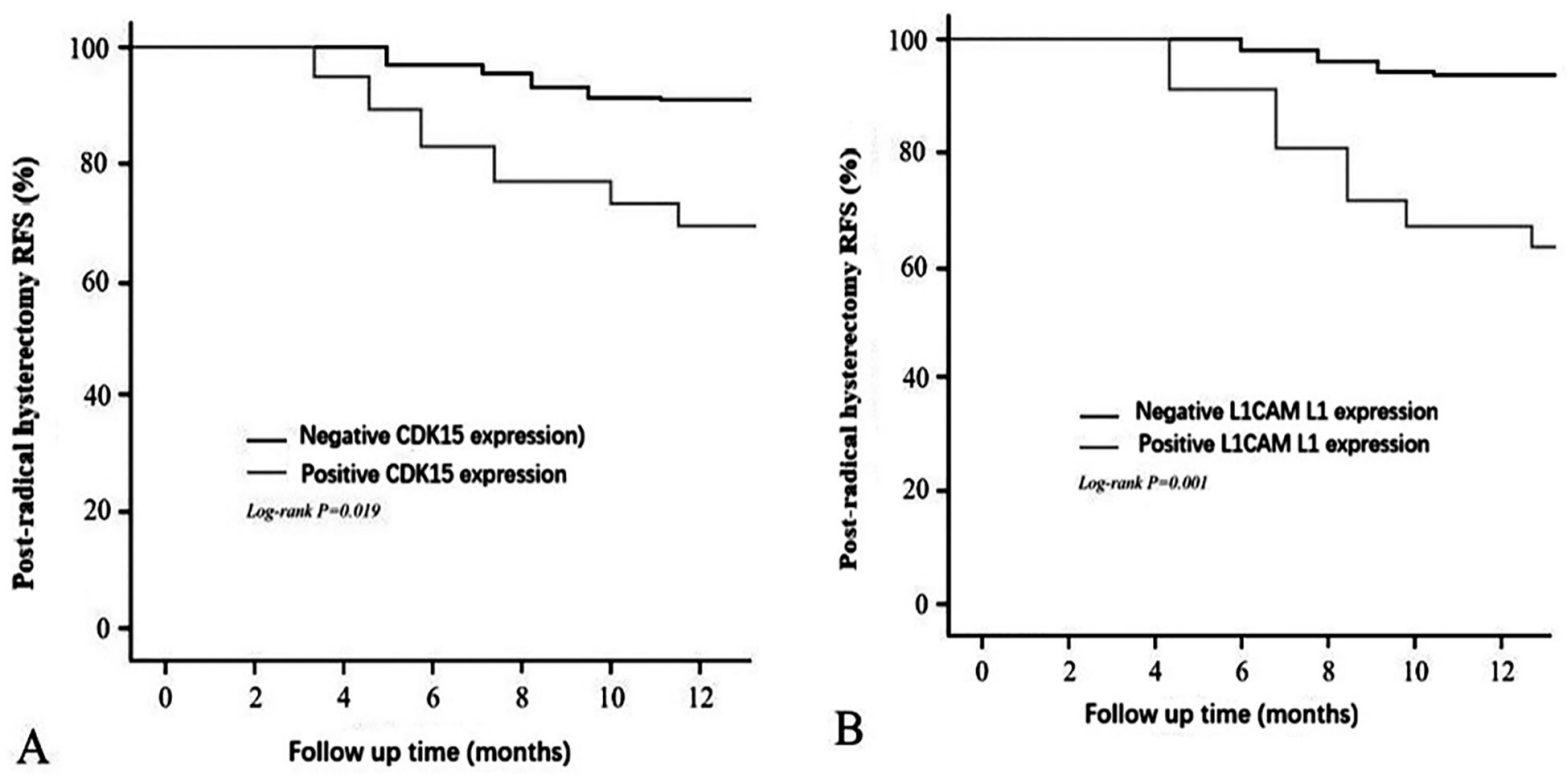
Recurrence and Metastasis of Cervical Cancer Patients After Radical Hysterectomy Stratified by CDK15 and L1CAM Expression.

The 12 months post-radical hysterectomy RFS rate was approximately 68.29% in cervical cancer patients with positive CDK15 expression and 63.16% in those with positive L1CAM L1 expression, both significantly lower than the corresponding 89.74% (negative CDK15) and 92.86% (negative L1CAM L1 expression), respectively(*Log-rank* P=0.019 and 0.001) Fig.1A and 1B.

FIGO stage (IIA-IIB), lymph node metastasis, lymphovascular invasion, positive CDK15 expression, and positive L1CAM L1 expression emerged as independent predictors for recurrence and metastasis after radical hysterectomy for cervical cancer (all P<0.05) Table-IV and Table-V.

**Table-IV:** Clinicopathological Characteristics of Recurrence/Metastasis Group and Non-Recurrence/Non-Metastasis Group (n, %).

Clinicopathological Characteristic	Number of Cases	Recurrence/Metastasis Group (n=17)	Non-Recurrence/ non-metastasis (n=63)	χ^2^	P
Age				0.448	0.503
≥50 years	32	8 (47.06)	24 (38.10)		
<50 years	48	9 (0.448)	39 (0.503)		
FIGO stage				5.410	<0.05
IA~IB	56	8 (47.06)	48 (76.19)		
IIA~IIB	24	9 (52.94)	15 (23.81)		
Pathological type				1.803	0.179
Adenocarcinoma	25	5 (29.41)	30 (47.62)		
Squamous cell carcinoma	55	12 (70.59)	33 (52.38)		
Maximum tumor diameter				0.256	0.613
≥4 cm	38	9 (52.94)	29 (46.03)		
<4 cm	42	8 (47.06)	34 (53.97)		
Histological differentiation degree				0.077	0.782
Poor differentiation	26	6 (35.29)	20 (31.75)		
Moderate to high differentiation	54	11 (64.71)	43 (68.25)		
Lymph node metastasis				8.416	<0.05
Yes	32	12 (70.59)	20 (31.75)		
No	48	5 (29.41)	43 (68.25)		
Lymphovascular invasion				7.265	<0.05
No	38	13 (76.47)	25 (39.68)		
No	42	4 (23.53)	38 (60.32)		
CDK15 expression				5.496	<0.05
Positive	41	13 (76.47)	28 (44.44)		
Negative	39	4 (23.53)	35 (55.56)		
L1CAM				10.515	<0.05
Positive	38	14 (82.35)	24 (38.10)		
Negative	42	3 (17.65)	39 (61.90)		

**Table-V:** Independent Predictors of Recurrence and Metastasis After Radical Hysterectomy for Cervical Cancer.

Variable	β	SE	Waldx^2^	P	HR	95%CI
FIGO staging (IIA~IIB)	0.592	0.533	6.064	<0.05	1.352	1.084~2.080
Lymph node metastasis	0.627	0.512	6.017	<0.05	1.559	1.108~2.514
Lymphovascular invasion	0.619	0.524	6.157	<0.05	1.495	1.059~2.412
Positive CDK15	0.604	0.526	6.192	<0.05	1.359	1.088~2.024
Positive L1CAM	0.632	0.502	6.275	<0.05	1.638	1.112~2.699

## DISCUSSION

The findings of this study demonstrated that the positive rates of CDK15 and L1CAM L1 expression in cervical cancer tissues were approximately 51.25% and 47.50%, respectively-significantly higher than the corresponding 22.50% and 18.75% in adjacent non-cancerous tissues(all P<0.05). This finding suggests that CDK15 and L1CAM L1 expression are upregulated in the early stages of cervical carcinogenesis, consistent with their expression patterns in solid tumors (e.g., lung cancer, breast cancer) as reported in prior investigations. As a key regulator of the cell cycle, CDK15 accelerates G1/S phase transition and suppresses cellular apoptosis by phosphorylating the Rb-E2F axis and activating the Wnt/β-catenin pathway; its aberrant activation disrupts cell cycle checkpoints, thereby fueling the proliferation of tumor cells.^7^ A prior study^8^ further demonstrated via fundamental experimental studies that the HPV-associated protein E6 creates a favorable microenvironment for CDK15 upregulation by inhibiting key regulatory factors, including p53 and Rb. In contrast, L1CAM primarily induces epithelial-mesenchymal transition (EMT) via signaling pathways including PI3K/Akt and MAPK, reducing intercellular adhesion and promoting tumor cell detachment from the primary tumor. Multiple studies have confirmed that high L1CAM L1 expression in solid tumors (e.g., lung cancer, breast cancer) is associated with the invasive and metastatic potential of these tumors.^9^,^10^

Upon analyzing differences in CDK15 and L1CAM L1 expression across cervical cancer patients stratified by clinicopathological characteristics, this study identified that CDK15 expression exhibited associations with FIGO stage, histological differentiation, lymph node metastasis, and lymphovascular invasion (all P<0.05). This suggests that aberrant CDK15 expression may be closely linked to the progression and invasive capacity of cervical cancer. A more advanced FIGO stage and lower histological differentiation correlated with a higher CDK15 positive rate; this could be attributed to CDK15 overexpression accelerating cell cycle progression, promoting malignant proliferation and invasion of tumor cells, and thereby facilitating tumor progression from early to advanced stages.^11^,^12^ High CDK15 expression in cervical cancer patients with lymph node metastasis or lymphovascular invasion suggests that CDK15 may be involved in lymphatic and hematogenous metastasis of tumor cells-a mechanism that mirrors its role in promoting metastasis via EMT modulation in lung cancer.^13^,^14^

In contrast, L1CAM L1 expression was correlated with FIGO stage, maximum tumor diameter, and lymph node metastasis(all P<0.05), indicating that high L1CAM L1 expression is linked to the local progression and lymph node metastatic potential of cervical cancer. The L1CAM L1 positive rate was higher in cervical cancer patients with a maximum tumor diameter ≥4 cm, suggesting that L1CAM may enlarge the primary tumor volume by enhancing the invasive capacity of tumor cells. Furthermore, the association between L1CAM L1 expression and cervical cancer lymph node metastasis further supports its role as a metastasis-associated molecule: L1CAM binds to molecules such as integrin αvβ3 in the extracellular matrix, mediating tumor cell crossing of the basement membrane of blood or lymphatic vessels to form metastatic foci. This mechanism has been validated in studies on metastasis in colorectal cancer and endometrial cancer.^15^ Synthesizing these findings, we reasonably hypothesize that CDK15 may provide a cytodynamic foundation for L1CAM L1-mediated invasion and migration via cell cycle regulation, with the two molecules acting synergistically to accelerate cervical cancer progression.

Kaplan-Meier analysis in this study revealed that the 12 months RFS following radical hysterectomy was approximately 68.29% in cervical cancer patients with positive CDK15 expression and 63.16% in those with positive L1CAM L1 expression-both significantly lower than the corresponding 89.74% (CDK15-negative) and 92.86% (L1CAM L1-negative), respectively (*Log-rank* P=0.019 and 0.001). This finding underscores the value of CDK15 and L1CAM L1 expression in predicting recurrence and metastasis following radical hysterectomy for cervical cancer. Recurrence or metastasis within 12 months postoperatively typically indicates more aggressive tumor biology; CDK15 and L1CAM L1 may enhance the proliferative activity and metastatic capacity of tumor cells, thereby contributing to early recurrence or distant metastasis in cervical cancer patients.^16^,^17^ Results from the Cox proportional hazards regression model further confirmed that positive CDK15 expression and positive L1CAM L1 expression are independent predictors of recurrence and metastasis following radical hysterectomy for cervical cancer(all P<0.05).

This implies that even after adjusting for clinicopathological characteristics, their expression status remains a valid independent indicator for assessing recurrence and metastasis risk post-radical hysterectomy-their predictive value is not confounded by traditional markers, facilitating more precise identification of high-risk patients. Based on these findings, for cervical cancer patients with positive CDK15 and/or L1CAM L1 expression, clinical strategies such as postoperative adjuvant chemoradiotherapy and intensified follow-up frequency may be considered to reduce recurrence and metastasis risk. Additionally, FIGO stage (IIA-IIB), lymph node metastasis, and lymphovascular invasion also independently impact recurrence and metastasis following radical hysterectomy for cervical cancer. We propose that CDK15 and L1CAM L1 may promote lymphovascular invasion, thereby leading to lymph node metastasis and establishing a “CDK15/L1CAM L1-lymphovascular invasion-lymph node metastasis-recurrence/metastasis” pathway that elevates post-radical hysterectomy recurrence and metastasis risk.^18^-^20^

### Limitations:

The follow-up duration was relatively short, precluding assessment of the relationship between CDK15/L1CAM L1 expression and long-term prognosis in patients post-radical hysterectomy.

## CONCLUSIONS

CDK15 and L1CAM L1 exhibit elevated expression in cervical cancer tissues, with their upregulation showing significant associations with advanced lesions, lymph node metastasis, and early postoperative recurrence/metastasis.

### Authors’ Contributions:

**JL:** Conceptualization, data collection, original draft, review and editing, and responsible for overall integrity of the study, and is responsible

**YS:** Supervision, conceptualization, methodology, and feedback on manuscript drafts.

**LC:** Writing - review and editing, and provision of frequent feedback on manuscript drafts.

**YA:** Formal analysis, data analysis, and software usage(SPSS). Critical Review.

**LS:** Review and provision of feedback on manuscript drafts.

All authors have read and approved the final manuscript.
